# Network structure impacts global commodity trade growth and resilience

**DOI:** 10.1371/journal.pone.0171184

**Published:** 2017-02-16

**Authors:** Ali Kharrazi, Elena Rovenskaya, Brian D. Fath

**Affiliations:** 1 Advanced Systems Analysis Program, International Institute for Applied Systems Analysis (IIASA), Laxenburg, Austria; 2 Graduate School of Public Policy, University of Tokyo, Tokyo, Japan; 3 Faculty of Computational Mathematics and Cybernetics, Lomonosov Moscow State University (MSU), Moscow, Russia; 4 Biology Department, Towson University, Towson, Maryland, United States of America; Cinvestav-Merida, MEXICO

## Abstract

Global commodity trade networks are critical to our collective sustainable development. Their increasing interconnectedness pose two practical questions: (i) Do the current network configurations support their further growth? (ii) How resilient are these networks to economic shocks? We analyze the data of global commodity trade flows from 1996 to 2012 to evaluate the relationship between structural properties of the global commodity trade networks and (a) their dynamic growth, as well as (b) the resilience of their growth with respect to the 2009 global economic shock. Specifically, we explore the role of network efficiency and redundancy using the information theory-based network flow analysis. We find that, while network efficiency is positively correlated with growth, highly efficient systems appear to be less resilient, losing more and gaining less growth following an economic shock. While all examined networks are rather redundant, we find that network redundancy does not hinder their growth. Moreover, systems exhibiting higher levels of redundancy lose less and gain more growth following an economic shock. We suggest that a strategy to support making global trade networks more efficient via, e.g., preferential trade agreements and higher specialization, can promote their further growth; while a strategy to increase the global trade networks’ redundancy via e.g., more abundant free-trade agreements, can improve their resilience to global economic shocks.

## Introduction

Global commodity trade is perhaps one of the most critical networks of our modern age. Given the expansion of globalization over the last centuries, trade networks have grown enormously in size and complexity. As reflected in the 17^th^ Sustainable Development Goal [1], to accommodate the growing population and to overcome poverty, the sustainable growth and development of global trade networks is essential beyond the current state. In leveraging the comparative advantage of production by various countries, flourishing trade is a fundamental basis for economic growth. Furthermore, food [2] and energy [3] security also become critically dependent on the reliable functioning of commodity trade networks.

Local or global shocks, such as economic and financial crises, political instability, and environmental disasters [4], can affect countries’ production or damage their exports and imports, causing financial losses for an exporter and supply deficits for an importer. Through internationally spread supply chains, a cascade of secondary negative effects can propagate throughout the entire trade network. A prominent example is the 2009 global economic crisis, which was reported to have led to long-term negative impacts on investments and trade needed for sustaining food and agricultural systems- especially in emerging and least developed countries [5].

Diversity in trade can help mitigate risks and enhance sustainability; however, overly connected commodity trade networks are prone to spreads of shocks. It is highly important, therefore, to explore which structures are favorable for limiting losses from shocks, while keeping conditions for short- and long-term growth, as well as which precautionary ex-ante measures can guide the commodity networks towards these structures.

Research leading to better management of highly interconnected global economic systems while in their early stage [6] emphasize the importance of system-level network properties for explaining these dynamics and informing grounded strategies and practices. The majority of research on international trade networks has focused on binary relationships, i.e., un-weighted flow links. Notably, an initial in-depth analysis of the world trade web (WTW) by [7,8] found that trade networks exhibit complex network properties of scale-free degree distribution, small-world properties, strong disassortativity, and clustering. These studies characterize world trade as an adjacency matrix and consider trade links as homogenous. However, this approach may not be realistic as actual import and export flows are heterogeneous and asymmetric in their intensity of connections. More recent studies examining world trade as weighted networks have found these networks to exhibit different properties than when examined through a binary approach. In particular, [9,10] have found that the WTW is only weakly disassortative and that countries characterized with relationships of higher trade intensity are more and not less clustered. However, these conclusions are difficult to translate into grounded practices and consequently new calls have emerged for network research approaches that can incorporate both the micro perspective of agency behavior and the macro perspective of a network’s structural dynamism [11]. Given the evolutionary history of survival of natural systems over centuries and millennia [12], new insights arising from our understanding of the resilience of ecological networks can be useful to understand the resilience of trade networks.

In this avenue, the WTW has been examined through the lens of extinction analysis and found to be resilient to random failures while fragile to targeted attacks and thus indicating that connectance, arising from globalization, has both positive and negative effects on network resilience [13]. The WTW has also been examined through the lens of evolutionary theory whereby it was found that decreases in the modularity of trade networks decreases their resilience to shocks [14]. In this paper, we examine the WTW through the ecological information-based approach, developed initially for analyzing the resilience of food webs [15], and emphasize the importance of the ratio between the system redundancy and efficiency for resiliency in the system. In this approach, a redundant network is one with a multiplicity of pathways to deliver flows, giving a higher probability of flow between any two randomly chosen nodes, whereas an efficient network is one that is articulated in a manner that restricts and constrains the flow choices. In this manuscript, for the first time, we demonstrate empirically how the system redundancy and efficiency are related to the ability of the global trade networks to ensure their resilience of growth in the face of a financial crisis while sustaining both long- and short-term growth.

We use the global commodity trade data from 1996 to 2012 to study both their long- and short-term growth, as well as to examine their resilience of growth to the global economic shock of 2009. Using global commodity trade data from UN Comtrade (http://comtrade.un.org/db/), an import-export database of the United Nations Statistics Division, in conjunction with complex network analysis, we highlight how systems-level network properties can reveal the success of explicit strategies which trade associations, e.g., national and international chambers of commerce and specialized commodity associations, can apply to promote growth and enhance the resilience of commodity trade growth. The 2009 global shock was chosen as it is viewed as the biggest economic crisis experienced in our modern global era and also because of the higher availability and quality of commodity trade data surrounding this year. While our results are derived from the 2009 global economic crisis, we anticipate that this approach may be applicable to other shocks and stresses in trade networks and also has a potential to contribute to emerging empirical research on resilience in other fields.

## Theoretical framework: Measuring network properties of efficiency and redundancy

The ecological information-based approach is a framework for identifying holistic properties based on an analysis of the network flows of material, energy, or information. While this approach has been developed in the ecological discipline [16,17], given the significant topological and dynamic similarities in all flow systems [18,19], it has been utilized in urban management [20,21] and more recently for research focusing on economic systems [19,22,23]. In this paper, we present a novel application for analyzing the growth and resilience of growth in global commodity trade networks. The underlying assumption within this approach is that growth and development are two fundamentally distinct processes of systems. While growth reflects the extensive property, e.g., the size of the system as total system throughput (TST), development on the other hand implies organizational and intensive properties of a system. In this approach the development of systems is based on two dialectically related system-level variables of network efficiency and redundancy.

While systems in the long-term exhibit a potential to increase their efficiency at the expense of redundancy [23,24], the relative dominance of these two system variables varies based on the system’s environmental constraints and decisions of its agents. The efficiency of a system reflects the degree of articulation or constraints to network flows. Efficiency tends to increase naturally in systems where agents select preferential interactions with other agents using a combination of competition and cooperation to develop pathways with higher intensity and specialization of resource flows. In global trade systems, preferential interactions are largely determined by locational proximity, cultural links, and strategic partnerships (supported for example by free-trade agreements). In the network approach employed here [25,26], the average mutual information is used to define the network efficiency of a system as follows:

$$\text{Efficiency}=\sum_{i,j}\frac{T_{ij}}{T_{..}}log\frac{T_{ij}T_{..}}{T_{i.}T_{.j}}$$ (Eq. 1)

Here, *T*_*ij*_ is a flow from agent *i* to agent *j*, *T*_*i*._ = ∑_*j*_*T*_*ij*_ is the total flow leaving agent *i*, *T*_.*j*_ = ∑_*i*_*T*_*ij*_ is the total flow entering agent *j* and the sum of all flows in the system, *T*_.._ = ∑_*ij*_*T*_*ij*_, is known as the Total System Throughput (*TST*).

The redundancy of a system reflects the degree of freedom or overhead in the network flows. Redundancy is exhibited as the diversity of pathways and is critical for a system’s capacity for innovation and ability to adapt to changing environmental conditions arising from shocks and disturbances. In global trade systems, the ability to choose from different agents of supply and demand is central to free market principles and enables maneuverability in supply. In the ecological information-based approach, conditional entropy is used to define the network redundancy [25] of a system as follows:

$$\text{Redundancy}=-\sum_{\text{i},\text{j}}\frac{{\text{T}}_{\text{ij}}}{{\text{T}}_{..}}\text{log}\frac{{\text{T}}_{\text{ij}}^{2}}{{\text{T}}_{\text{i}.}{\text{T}}_{.\text{j}}}$$ (Eq. 2)

Intuitively, systems need to balance these opposing properties. For example, HS40, the ‘Rubber and articles thereof’ commodity network is highly redundant (16 year average efficiency of 0.6622 bits and redundancy of 5.6326 bits); while HS01, the ‘Live animals’ commodity network is highly efficient (16 year average efficiency of 1.7726 bits and redundancy of 2.9934 bits). Overly redundant networks may be stagnant and lack the efficiency to grow, while overly efficient networks may be brittle and prone to collapse when subjected to stress. To help determine a balance between constraints imposed by efficiency and the flexibility provided by redundancy, the relative order α in the system is introduced as:

α=Efficiency/(Efficiency+Redundancy),where0≤α≤1 (Eq. 3)

The ratio α is a convenient way to express the degree of which property dominates the system at a given time. Departing from the relative order and invoking the Boltzmann measure [27] of its disorder, the level of a system’s Theoretical Resilience can be expressed as [22]:

Theoretical Resilience=−αlog(α) (Eq. 4)

From Eq 4, a maximum value for Theoretical Resilience is derived as 1/e ≈ 0.3704 (independent of the logarithm’s base). Empirical investigations have determined that natural networks, e.g., ecosystems and food webs, lie in close proximity to this maximum [25], while economic systems indicate higher levels of redundancy [23]. The maximum resilience value however, should be seen as a theoretical benchmark; optimal (minimal) vulnerability of real heterogeneous systems under various environmental conditions may be different from this value.

Theoretical Resilience (Eq 4) signifies the balance between efficiency and redundancy as a single metric and therefore is useful in exploring and comparing the configurations of heterogeneous networks. However, the analytical implications of the variable are limited and should be approached with caution. Firstly, the variable does not differentiate among shocks against to which the network system might be judged to be resilient. Secondly, despite arguments of bio-mimicry, derived from evolutionary observations [23], it may not be possible or desirable to prescribe an optimal level of Theoretical Resilience to socio-economic networks. Thirdly, changes to the levels of Theoretical Resilience are difficult to translate into actions, strategies, and practices by policy- and decision- makers at either the local or systems level.

### 2.1 Growth and resilience of growth to economic shocks

**Fig 1:**
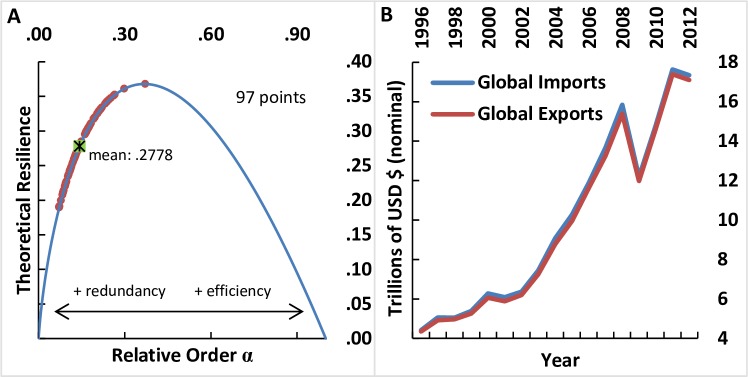
*A*) Average values of the relative order *α* and Theoretical Resilience for 97 commodity trade networks 1996–2012. *B*) Trend of global commodity imports *T*_.*j*_ and exports *T*_*i*._ between 1996 and 2012.

In this study, we examine the network configurations of global commodity trade sectors between the years of 1996–2012 using the ecological information-based approach. Our basic network diagnostics indicate that the 17-year averages of the global commodity networks, while over-redundant, lie rather close (mean of 0.2778 and standard deviation of 0.0424) to the Theoretical Resilience maximum (Fig 1*A*). In the commodity trade networks examined in this study, we generally observe that networks with higher efficiency tend to exhibit lower redundancy. Furthermore, it is enlightening to realize that in redundancy-dominated systems close to the Theoretical Resilience maximum, the value of Theoretical Resilience is defined mostly by efficiency, while redundancy plays a negligible role (see Fig A in S1 File).

In consideration of the above, we use the ecological information-based approach to examine empirically how the underlying network configurations affected the growth of trade and the resilience of growth in the face of global economic shocks. Here, we consider 2009 as an economic shock year based on the IMF criteria for a global recession [28], i.e., a decline in annual real world GDP per-capita (purchasing power parity weighted). In line with the IMF criteria, the global commodity trade data also indicate a significant drop in global imports and exports in 2009 (Fig 1*B*).

## Methodology: Constructing the global commodity trade network

The data used for this study are based on the United Nations Commodity Trade Statistics Database (UN-Comtrade). These data represent annual global trade detailed by commodities and partner countries in nominal US dollar values. Three main classification systems are provided in the UN-Comtrade data and here we use the Harmonized System (HS), introduced in 1988, which is a detailed breakdown of products to individual categories. Given this detailed product breakdown, the HS classification is more suitable for questions concerning tariffs, customs, and trade quotas.

**Fig 2:**
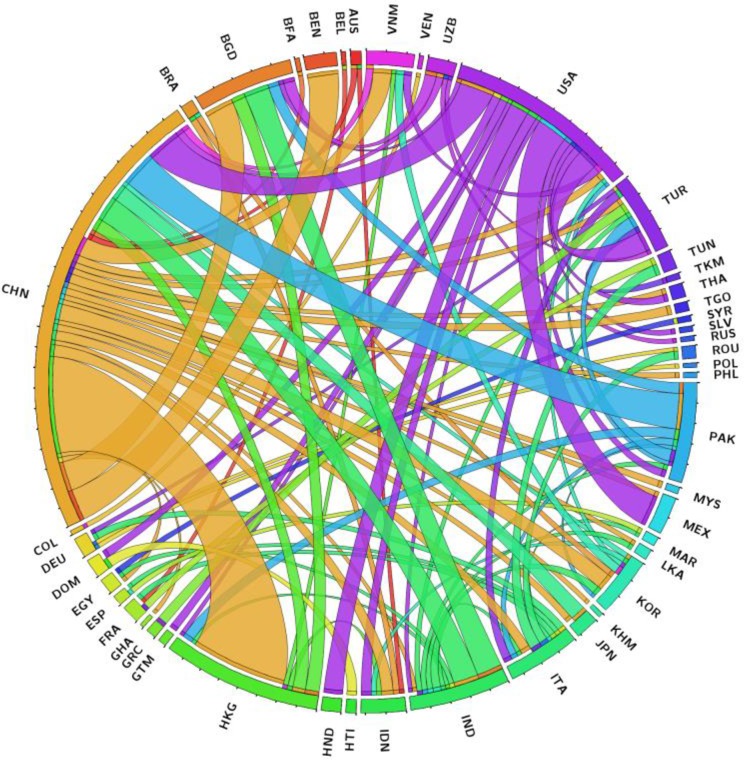
Global trade of cotton (HS 52) in 2009; only 49 countries with trade above $ 100 million USD are depicted. Names of countries are represented by the ISO 3166 standard three letter code.

We have considered the entire HS 1996 revision UN-Comtrade dataset for country-to-country trade of commodity goods in 97 classifications (see Table A in S2 File). Each of these classifications represents particular commodities traded globally through a densely interconnected network–as an example Fig 2 depicts the global trade of cotton in 2009. In total, we have analyzed 1,649 network–years (97 sectors from 1996 to 2012). Given that these datasets are an international effort, their compilation takes time and therefore the early years of 1992–1995 and most recent years 2013–2014 of the HS classifications were not considered, as the reported countries are not complete and therefore the datasets might be distorted. For a full list of available years please consult the UN-Comtrade website.

In the interest of repeating our results, the following technical issues should be mentioned. Firstly, it is not uncommon to see a directed trade flow reported twice in the database with a large difference between the two values, i.e., one by an importing country and one by an exporting country. This difference may be due to the fact that the data reported by the importer is based on Cost, Insurance, and Freight (CIF) accounting whereas the data reported by the exporter is based on Free-On-Board (FOB) accounting. With the assumption that imports are carefully scrutinized for taxing purposes and therefore more accurate, we give primacy to the importer’s reports when available. Secondly, we considered only trade between countries and territories in our compilation; data from areas not elsewhere specified (NES), specialized categories, free zones, bunkers, and data from the reporter to itself were not considered. Thirdly, the reported flows are based on nominal US dollar values and therefore were adjusted to real values using US dollar Commodity Price Index (CPI) inflation data from the U.S. Bureau of Labor Statistics. Fourthly, the data sources reflect aggregate level trade flows of commodities and their immediate by-products, but not on embodied commodities.

## Results and discussions

### 4.1 What network properties are conducive to commodity trade growth?

The differences in the growth of commodity trade may be explained by examining their underlying network configurations. We examine correlations between the long- and short-term sectorial TST growth rates and corresponding commodity trade network configurations using two key network indicators of efficiency (Eq 1) and redundancy (Eq 2). For the long-term, we use the average TST growth rates over the 1997–2012 period and average network configurations over the same period. For the short-term, we use 3-, 5-, and 7-year moving averages of both TST growth rates and network configurations. The TST growth rate (gr) for sector (s) at time (t) is defined as: $gr_{t}^{s}={(TST}_{t}^{s}-TST_{t-1}^{s})/{TST}_{t-1}^{s}$ and the *τ*-year moving average (*MA*_*τ*_) TST growth rate for sector (s) at time (t) is defined as: ${MA}_{t,\tau }^{s}=\left(gr_{t-\tau }^{s}+{gr_{t-\tau +1}^{s}+..+gr}_{t}^{s}\right)/\tau \;(\tau =1,3,5,7)$. The 3-sigma rule [29] was applied to remove outliers in the correlation data. We use Spearman’s rank correlation coefficient to detect any statistically significant monotonic relationship between the two examined variables and Pearson’s correlation coefficient to detect any linear relationship.

**Fig 3:**
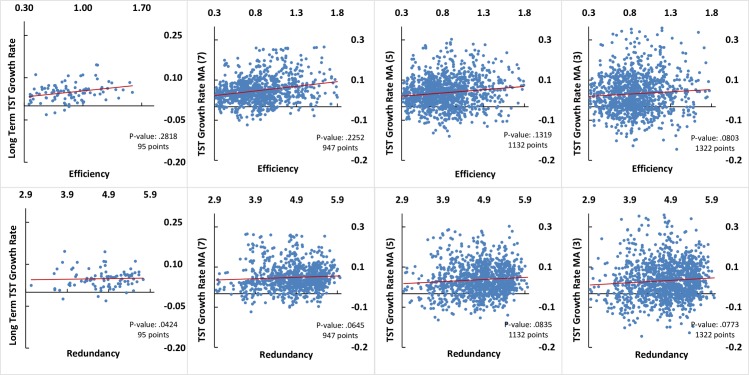
Long-term average and short-term moving average (3, 5, and 7 years) sectorial TST growth rates and corresponding network properties of efficiency and redundancy and significance of the paired Pearson's correlations. Number of points, 16-year: 95, MA(7): 947, MA(5): 1132, MA(3): 1322.

In examining long- and short-term growth rates and efficiency, the Spearman’s rank and Pearson’s correlations reveal a strong positive correlation. The Spearman’s rank and Pearson’s correlations reveal no correlation between long-term growth rates and redundancy, while revealing a strong positive correlation between short-term growth rates and redundancy (Table 1 and Fig 3).

**Table 1:** Spearman’s rank and Pearson’s correlations between long-term growth rates (16-year average, number of points 95) and short-term growth rates (moving average (MA) year, number of points MA(7): 947, MA(5): 1132, MA(3): 1322) and corresponding network properties of efficiency and redundancy.

Pairs of Correlated Variables	Spearman's Coeff. (P-value)	Pearson’s Coeff. (P-value)
Long-term TST growth rate & Efficiency	**0.2189** (0.0330)	**0.2818** (0.0057)
TST growth rate MA(7) & Efficiency	**0.1930** (0.0000)	**0.2252** (0.0000)
TST growth rate MA(5) & Efficiency	**0.1231** (0.0000)	**0.1319** (0.0000)
TST growth rate MA(3) & Efficiency	**0.0643** (0.0194)	**0.0803** (0.0035)
Long-term TST growth rate & Redundancy	**0.0925** (0.3726)	**0.0424** (0.6832)
TST growth rate MA(7) & Redundancy	**0.1202** (0.0002)	**0.0645** (0.0471)
TST growth rate MA(5) & Redundancy	**0.0969** (0.0011)	**0.0835** (0.0049)
TST growth rate MA(3) & Redundancy	**0.0918** (0.0008)	**0.0773** (0.0049)

Additionally, we use multiple regression analysis to examine if the TST growth rates (both long- and short-term) can be explained better if both independent variables of efficiency and redundancy are taken into consideration simultaneously. These investigations reveal no additional insights regarding the relation between the long-term average and short-term moving average (3, 5, and 7 years) sectorial TST growth rates and corresponding network properties of efficiency and redundancy (Table 2).

**Table 2:** Multiple regressions between long-term and short-term moving average sectorial TST growth rates (dependent variables) and network properties of efficiency and redundancy (independent variables). Y = a + a_0_Efficiency + a_1_Redundancy. Number of points for long-term average: 97, MA(7): 970, MA(5): 1164, MA(3): 1358.

Dependent variable—Y	a_0_ (p-value)	a_1_ (p-value)
Long-term TST growth rate	**0.0263** (0.0205)	**0.0039** (0.4846)
TST growth rate MA(7)	**0.0369** (0.0000)	**0.0040** (0.1500)
TST growth rate MA(5)	**0.0360** (0.0000)	**0.0057** (0.0704)
TST growth rate MA(3)	**0.0331** (0.0000)	**0.0047** (0.1894)

These results indicate 1) a significant positive correlation between the network efficiency and both long- and short-term commodity trade growth, 2) no significant correlation between redundancy and long-term growth, and 3) a significant positive correlation between redundancy and short-term commodity trade growth. The role of redundancy here may be attributed to the fact that commodity trade networks are over-redundant. Therefore, networks with higher efficiency tend to achieve higher levels of growth, while, despite possible contrary assumptions, higher network redundancy in the short-term does not hinder growth rates.

### 4.2 How do network properties affect the resilience of growth to economic shocks?

The configurations of global commodity trade networks may significantly affect their vulnerability to loss of growth following an economic shock. Considering the 2009 global economic shock year, we define here the Vulnerability Index (VI) of a commodity trade network as the difference between its 3-year average pre-shock and post-shock growth rates. We further dichotomize networks with a positive VI value as ‘gainers’ and those with a negative value as ‘losers’.

To ensure statistical quality and minimize the influence of random variables, the VI is based on the difference between the 3-year average pre-shock and post-shock growth rates ($\bar{gr}$) of the commodity sector (s) as:

$${VI^{s}=(\bar{gr})}_{2010,2011,2012}-{\left(\bar{gr}\right)}_{2006,2007,2008}$$ (Eq. 5)

and the dichotomized VI of commodity sector (s) as:

$$VI^{s}=\{{}^{\prime }gainer^{\prime }\;if\;VI^{s}\geq 0\;\&\;{}^{\prime }loser^{\prime }\;if\;VI^{s}<0\}$$ (Eq. 6)

Based on the above definitions, we explore how network configurations are related to whether a particular commodity network appears as a ‘gainer’ or ‘loser’ after the shock. The 3-sigma rule was applied to remove two outlier commodity sectors, i.e., HS1 and HS99 sector (see Table A in S2 File). For this purpose, we use the point-biserial correlation [30] to test for a relationship between a dichotomous VI variable and the pre-shock 3-year average network configurations.

The point-biserial coefficient is calculated as: $r=({\bar{VI}}_{1}^{S}-{\bar{VI}}_{0}^{S})\sqrt{p(1-p)}/S_{{VI}^{s}}$, where, ${\bar{VI}}_{0}^{S}$ is the mean of *VI*^*s*^ when *VI*^*s*^ < 0, ${\bar{VI}}_{1}^{S}$ is the mean of *VI*^*s*^ when *VI*^*s*^ ≥ 0, $S_{{VI}^{s}}$ is the standard deviation of *VI*^*s*^, and *p* is the proportion of values where *VI*^*s*^ ≥ 0.

This result of this analysis, as depicted in Table 3, reveals a significant negative correlation between the dichotomized VI and the pre-shock 3-year average efficiency and no significant correlation with the pre-shock average redundancy.

**Table 3:** Point-biserial correlations between VI and pre-shock 3-year averages of efficiency and redundancy.

Pairs of Correlated Variables	Point-biserial Coeff. (r)	P-value
Efficiency vs. *VI*	**−0.2836**	0.0049
Redundancy vs. *VI*	**−0.0105**	0.9186

**Fig 4:**
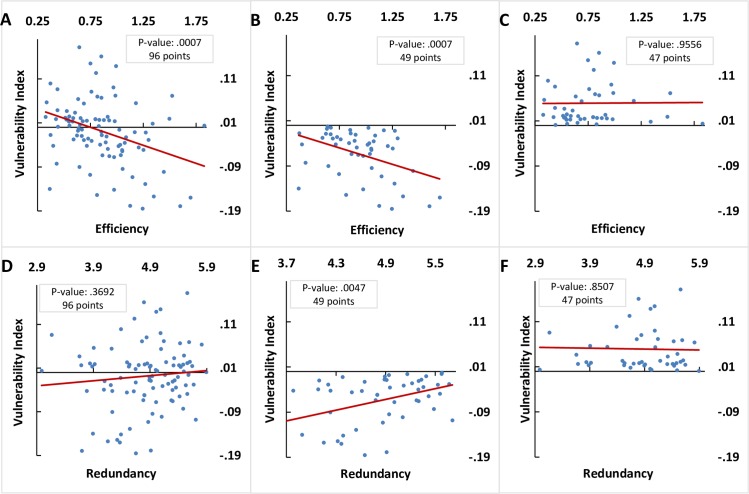
VI values and corresponding 3-year pre-shock averages of efficiency and redundancy and significance of the paired Pearson's correlations. Total VI values (*A*, *D*), negative VI values (*B*, *E*), and positive VI values (*C*, *F*).

In order to further explore the relation between the impact of the shock and network configurations, we examine Spearman’s rank and Pearson’s correlations between the VI and the pre-shock 3-year average of efficiency and redundancy. We find a significant negative correlation between the VI and average efficiency and no significant correlation between VI and average redundancy (Fig 4*A and* 4*D* and Table 4). These results warrant a further examination of the networks categorized as ‘loser’ and ‘gainer’ and their separate relationship with the pre-shock network configurations. For ‘loser’ networks we find that their VI is negatively correlated with the average 3-year pre-shock average of efficiency and positively correlated with the 3-year pre-shock average of redundancy (Fig 4*B and* 4*E* and Table 4). For ‘gainer’ networks, the VI is not found to be correlated with the pre-shock 3-year average of redundancy an efficiency (Fig 4*C and* 4*F* and Table 4).

**Table 4:** Spearman’s rank and Pearson’s correlations between VI and pre-shock 3-year averages of efficiency and redundancy. Number of points, ***VI***^***s***^: 95, ***VI***^***s***^ < **0**: 49, ***VI***^***s***^ > **0**: 46.

Pairs of Correlated Variables	Spearman's Coeff. (P-value)	Pearson’s Coeff. (P-value)
Efficiency vs. *VI*^*s*^	**−0.3791** (0.0002)	**−0.3625** (0.0003)
Efficiency vs. *VI*^*s*^ < 0	**−0.3892** (0.0057)	**−0.4127** (0.0032)
Efficiency vs. *VI*^*s*^ > 0	**0.1011** (0.5039)	**0.0881** (0.5603)
Redundancy vs. *VI*^*s*^	**0.0929** (0.3703)	**0.1028** (0.3216)
Redundancy vs. *VI*^*s*^ < 0	**0.3502** (0.0136)	**0.3976** (0.0047)
Redundancy vs. *VI*^*s*^ > 0	**−0.1064** (0.4816)	**−0.0881** (0.5606)

We use the Chow test [31] to confirm that the network configurations of commodity trade networks are indeed qualitatively different between ‘loser’ and ‘gainer’ networks. The Chow test is defined as: $\frac{\left(S_{{VI}^{S}}-(S_{VI^{s}\geq 0}+S_{VI^{s}<0}))/(k\right)}{\left(S_{VI^{s}\geq 0}+S_{VI^{s}<0})/(N_{VI^{s}\geq 0}+N_{VI^{s}<0}-2k\right)}$ where, $S_{{VI}^{S}}$ are the sum of squared residuals from the combined groups, $S_{VI^{s}\geq 0}$ is the sum of the squared residuals from the ‘gainer’ group, and $S_{VI^{s}<0}$ are the sum of the squared residuals from the ‘loser’ group. $N_{VI^{s}\geq 0}$ and $N_{VI^{s}<0}$ are the number of observations in each group and *k* is the total number of parameters. The Chow test statistics follows the F-distribution with *k* and $N_{VI^{s}\geq 0}+N_{VI^{s}<0}-2k$ degrees of freedom.

Results of the Chow test statistic indicate that a structural break indeed exists and confirms the hypotheses that the independent variables of efficiency and redundancy maintain different impacts on the dependent variable of VI [Efficiency: F = 33.8928 and Redundancy: F = 41.0834, where F-critical (3, 91) ~ P (0.05) = 2.7047]. Therefore, networks identified as ‘gainer’ or ‘loser’ are impacted differently from their pre-shock efficiency and redundancy levels.

These results indicate 1) commodity networks with higher levels of efficiency before an economic shock are more likely to be losers of growth following an economic shock and 2) these networks lose less growth if they had maintained lower levels of efficiency and consequently higher levels of redundancy. Therefore, economic shocks impact networks differently based on their configurations; higher efficiency makes commodity networks less resilient in growth following an economic shock.

## Summary and conclusion

Evolutionary and ecological traits may advance our ability to situate the concept of resilience to our social, environmental, and economic activities and increase our capacity for sustainable development. This requires an assessment of the usefulness of the concept of resilience through empirically testable theory. In this manuscript, we demonstrate how the ecological information-based approach can reveal network properties that significantly affect growth and the resilience of growth to shocks. Similar to natural ecological systems, commodity trade networks exhibit higher growth with higher levels of efficiency and greater resilience of growth to shocks with higher levels of redundancy. This confirms a fundamental tradeoff between efficiency and growth, on one hand, and redundancy and resilience of growth on the other. While in this paper, we discuss network configurations favorable to growth and resilience of growth based on correlations, given more detailed data availability, e.g., monthly trade data, questions of causality can also be addressed in future research.

The properties of efficiency and redundancy are system-level structural configurations which emerge from the metabolic flows of the network. Network flows quantify exchanges between compartments which only implicitly reflect the various social and economic behavioral drivers of these compartments at the agency level. Therefore, it may be challenging to modify a network’s structure through behavioral incentives at the agency level. However, to translate our findings to grounded applications one must not focus on either agency or structure but accept their mutual dependency. While taking into consideration the challenges of this duality [32], we see a central role for trade associations in increasing awareness and promoting grounded practices and strategies based on the network properties of efficiency and redundancy for achieving growth and resilience of growth to economics shocks.

Global commodity trade networks constitute complex interactions between private and public organizations, including governments, manufacturers, consumers, shippers, insurance companies, and logistic service providers. A multi-stakeholder initiative including these actors is best achieved through trade associations, e.g., national and international chambers of commerce and specialized commodity sector associations. Individual economic agents operating in the same market establish trade associations to represent and provide services to them at a systems-level which is of collective benefit. These trade associations’ *raison d’etre* is to formulate strategies and practices that support what economists call ‘the public goods’, i.e., challenges that improve collective well-being but unlikely to be undertaken by individual economic agents. The basic functions of trade associations include collective bargaining, development of standard practices, dissemination of best-practices, and market influence.

In this vein, trade associations can increase redundancy by promoting and developing contingency plans for the diversification of trade among suppliers and consumers or conversely increase efficiency by promoting vertical integration and focused supply chain networks. Redundancies can also be enhanced through the diversification of production among more countries and lead to higher export and import flows. While such strategies are well known in the operations management literature [33], the impacts of the implementation of these strategies, their trade-offs, and associated costs have been difficult to measure. The ecological information-based approach provides objective measurements which can help evaluate the cost, benefit, and appropriate balance of available strategies. To improve the precision of these strategies, more research is required in quantifying the impact of economic shocks on trade growth. This may be achieved by examining past experiences to economic shocks and by increasing future visibility through dataset at higher temporal granularities, e.g., quarterly, monthly, and real-time data.

Given the highly interconnected nature of today’s social, economic, and environmental networks, our sustainable development is made more vulnerable to globally adverted shocks. Therefore, it is critical to advance our empirical understanding of the effect of network structure on growth and resilience of growth. The ecological information-based approach offers an important empirical method for evaluating network resilience. In this paper, we applied this approach to commodity trade networks and empirically tested how the network properties of redundancy and efficiency can be developed into grounded strategies for growth and resilience of growth to economic shocks. Resiliency of growth, however, is not necessarily a sustainable solution; it is an assessment of the given economic paradigm, not a statement of the desired, and the merits of continued trade growth should be evaluated based on the three economic, environmental, and social pillars of sustainable development. For future research we suggest an examination of the overlaps and tradeoffs of redundancy and efficiency with other network properties found in ecological systems, e.g. modularity and tightness of feedback loops [34], relevant to resilience. We also hope the work presented in this paper will encourage further research in examining other critical networks relevant to sustainable development, e.g., the water, food, and energy nexus, and in advancing the ecological information-based approach by rendering it more communicable to policy makers and making it more reliable as a methodological tool.

## Supporting information

S1 File Theoretical Resilience as a function of efficiency and redundancy. (DOCX)

S2 File Table of Harmonized System commodity classifications. (DOCX)
