# Supplementary material for: Network structure impacts global commodity trade growth and resilience
Source: PLoS One. 2017 Feb 16;12(2):e0171184. doi: 10.1371/journal.pone.0171184 (PMC5312938; doi:10.1371/journal.pone.0171184)
Supplement: S1 File — (DOCX) [file pone.0171184.s001.docx]

**Supporting Information**

**S1. Theoretical Resilience as a function of efficiency and redundancy**

The Theoretical Resilience indicator is determined through the relation between efficiency and redundancy in a system. However, the contribution of efficiency and redundancy in determining the Theoretical Resilience of a system varies depending on these two values. Below we demonstrate that in over-redundant networks, close to the maximum Theoretical Resilience, the Theoretical Resilience of a system is determined more by the value of efficiency. Conversely, in over-efficient networks, close to the theoretical maxim, the opposite is valid, where the value of Theoretical Resilience is determined more by the value of redundancy. Consider $Theoretical Resilience$ as a function defined by efficiency (E) and redundancy (R):

$Theoretical Resilience (E,R)=-\alpha Log_{b}\alpha$ , where $b>1 \mathrm{and} \alpha= \frac{E}{E+R}$

We can then examine the change of Theoretical Resilience relative to the change of efficiency and redundancy as by evaluating the respective partial derivatives:

$\frac{\partial Theoretical Resilience \left( E,R \right)}{\partial E}=\frac{R}{{(E+R)}^{2}} Log_{b}\frac{E+R}{eE}$

$\frac{\partial Theoretical Resilience \left( E,R \right)}{\partial R}=\frac{E}{{(E+R)}^{2}} Log_{b}\frac{eE}{E+R}$

Consider over-redundant systems, where $\alpha<\frac{1}{e}$. In this case $Log_{b}\frac{E+R}{eE}>Log_{b}1=0$. Hence, $\frac{\partial Theoretical Resilience \left( E,R \right)}{\partial E}>0$. Using the Taylor's expansion around $x=1$, we have for $x\in(0,1)$: $\log_{b} x\approx\frac{x-1}{\ln b}.$ Thus we can deduce that: $\frac{\partial Theoretical Resilience \left( E,R \right)}{\partial R}\approx\frac{eE}{{(E+R)}^{2}}\frac{\frac{E}{E+R}-\frac{1}{e}}{\ln b}$ *.* Therefore, if an over-redundant system exhibits values close to the maximum Theoretical Resilience, i.e., if $\frac{E}{R+E}\approx\frac{1}{e}$ , then $\frac{\partial Theoretical Resilience \left( E,R \right)}{\partial R}<0$ and is close to zero.

Conversely, in over-efficient systems, where $\alpha>\frac{1}{e}$, if a system exhibits values close to the maximum Theoretical Resilience, i.e., if $\frac{E}{R+E}\approx\frac{1}{e}$ , then $\frac{\partial Theoretical Resilience \left( E,R \right)}{\partial E}<0$ and is close to zero.

We illustrate the above, by generating Theoretical Resilience values as a function of random values of efficiency and redundancy in both over-redundant (Fig A) and over-efficient (Fig B) systems. In examining Fig A, it is evident that in over-redundant systems, there is no clear relation between redundancy and Theoretical Resilience while efficiency and Theoretical Resilience are positively related. Conversely, in the case of over-efficient system, as illustrated in Fig B, it is evident that there is no clear relation between efficiency and Theoretical Resilience while redundancy and Theoretical Resilience are positively related.

**Fig A.** Theoretical Resilience (horizontal axis) as a function of random values of efficiency (E) and redundancy (R) (vertical axis) in over-redundant networks where,$\alpha<\frac{1}{e}$

**Fig B.** Theoretical Resilience (horizontal axis) as a function of random values of efficiency (E) and redundancy (R) (vertical axis) in over-efficient networks where, $\alpha>\frac{1}{e}$

Our findings therefore indicate that while the Theoretical Resilience indicator is determined through the relation between the variables of efficiency and redundancy, Theoretical Resilience however is mostly determined by the variable in least supply. In over-redundant systems, Theoretical Resilience is determined more by efficiency and conversely in over-efficient systems theoretical resilience is determined more by redundancy. In the case of global commodity trade networks, where networks are over-redundant, theoretical resilience is therefore highly related to efficiency and depends less on redundancy.
