# Supplementary material for: Network structure impacts global commodity trade growth and resilience
Source: PLoS One. 2017 Feb 16;12(2):e0171184. doi: 10.1371/journal.pone.0171184 (PMC5312938; doi:10.1371/journal.pone.0171184)
Supplement: S2 File — (DOCX) [file pone.0171184.s002.docx]

**Supporting Information**

**S2. Table of Harmonized System commodity classifications**

| **HS.96 Code** | **Description** |
| --- | --- |
| 01 | Live animals |
| 02 | Meat and edible meat offal |
| 03 | Fish, crustaceans, molluscs, aquatic invertebrates nes |
| 04 | Dairy products, eggs, honey, edible animal product nes |
| 05 | Products of animal origin, nes |
| 06 | Live trees, plants, bulbs, roots, cut flowers etc |
| 07 | Edible vegetables and certain roots and tubers |
| 08 | Edible fruit, nuts, peel of citrus fruit, melons |
| 09 | Coffee, tea, mate and spices |
| 10 | Cereals |
| 11 | Milling products, malt, starches, inulin, wheat gluten |
| 12 | Oil seed, oleagic fruits, grain, seed, fruit, etc, nes |
| 13 | Lac, gums, resins, vegetable saps and extracts nes |
| 14 | Vegetable plaiting materials, vegetable products nes |
| 15 | Animal,vegetable fats and oils, cleavage products, etc |
| 16 | Meat, fish and seafood food preparations nes |
| 17 | Sugars and sugar confectionery |
| 18 | Cocoa and cocoa preparations |
| 19 | Cereal, flour, starch, milk preparations and products |
| 20 | Vegetable, fruit, nut, etc food preparations |
| 21 | Miscellaneous edible preparations |
| 22 | Beverages, spirits and vinegar |
| 23 | Residues, wastes of food industry, animal fodder |
| 24 | Tobacco and manufactured tobacco substitutes |
| 25 | Salt, sulphur, earth, stone, plaster, lime and cement |
| 26 | Ores, slag and ash |
| 27 | Mineral fuels, oils, distillation products, etc |
| 28 | Inorganic chemicals, precious metal compound, isotopes |
| 29 | Organic chemicals |
| 30 | Pharmaceutical products |
| 31 | Fertilizers |
| 32 | Tanning, dyeing extracts, tannins, derivs,pigments etc |
| 33 | Essential oils, perfumes, cosmetics, toileteries |
| 34 | Soaps, lubricants, waxes, candles, modelling pastes |
| 35 | Albuminoids, modified starches, glues, enzymes |
| 36 | Explosives, pyrotechnics, matches, pyrophorics, etc |
| 37 | Photographic or cinematographic goods |
| 38 | Miscellaneous chemical products |
| 39 | Plastics and articles thereof |
| 40 | Rubber and articles thereof |
| 41 | Raw hides and skins (other than furskins) and leather |
| 42 | Articles of leather, animal gut, harness, travel goods |
| 43 | Furskins and artificial fur, manufactures thereof |
| 44 | Wood and articles of wood, wood charcoal |
| 45 | Cork and articles of cork |
| 46 | Manufactures of plaiting material, basketwork, etc. |
| 47 | Pulp of wood, fibrous cellulosic material, waste etc |
| 48 | Paper & paperboard, articles of pulp, paper and board |
| 49 | Printed books, newspapers, pictures etc |
| 50 | Silk |
| 51 | Wool, animal hair, horsehair yarn and fabric thereof |
| 52 | Cotton |
| 53 | Vegetable textile fibres nes, paper yarn, woven fabric |
| 54 | Manmade filaments |
| 55 | Manmade staple fibres |
| 56 | Wadding, felt, nonwovens, yarns, twine, cordage, etc |
| 57 | Carpets and other textile floor coverings |
| 58 | Special woven or tufted fabric, lace, tapestry etc |
| 59 | Impregnated, coated or laminated textile fabric |
| 60 | Knitted or crocheted fabric |
| 61 | Articles of apparel, accessories, knit or crochet |
| 62 | Articles of apparel, accessories, not knit or crochet |
| 63 | Other made textile articles, sets, worn clothing etc |
| 64 | Footwear, gaiters and the like, parts thereof |
| 65 | Headgear and parts thereof |
| 66 | Umbrellas, walking-sticks, seat-sticks, whips, etc |
| 67 | Bird skin, feathers, artificial flowers, human hair |
| 68 | Stone, plaster, cement, asbestos, mica, etc articles |
| 69 | Ceramic products |
| 70 | Glass and glassware |
| 71 | Pearls, precious stones, metals, coins, etc |
| 72 | Iron and steel |
| 73 | Articles of iron or steel |
| 74 | Copper and articles thereof |
| 75 | Nickel and articles thereof |
| 76 | Aluminium and articles thereof |
| 78 | Lead and articles thereof |
| 79 | Zinc and articles thereof |
| 80 | Tin and articles thereof |
| 81 | Other base metals, cermets, articles thereof |
| 82 | Tools, implements, cutlery, etc of base metal |
| 83 | Miscellaneous articles of base metal |
| 84 | Nuclear reactors, boilers, machinery, etc |
| 85 | Electrical, electronic equipment |
| 86 | Railway, tramway locomotives, rolling stock, equipment |
| 87 | Vehicles other than railway, tramway |
| 88 | Aircraft, spacecraft, and parts thereof |
| 89 | Ships, boats and other floating structures |
| 90 | Optical, photo, technical, medical, etc apparatus |
| 91 | Clocks and watches and parts thereof |
| 92 | Musical instruments, parts and accessories |
| 93 | Arms and ammunition, parts and accessories thereof |
| 94 | Furniture, lighting, signs, prefabricated buildings |
| 95 | Toys, games, sports requisites |
| 96 | Miscellaneous manufactured articles |
| 97 | Works of art, collectors pieces and antiques |
| 99 | Commodities not elsewhere specified |

**Table A.** Harmonized System 1996 revision (HS 96) commodity classification codes and descriptions.
